# Information and Emotional Support Needs of Families Whose Infant Was Diagnosed With SCID Through Newborn Screening

**DOI:** 10.3389/fimmu.2020.00885

**Published:** 2020-05-06

**Authors:** Melissa Raspa, Molly Lynch, Linda Squiers, Angela Gwaltney, Katherine Porter, Holly Peay, Alissa Huston, Brian Fitzek, John G. Boyle

**Affiliations:** ^1^RTI International Research Triangle Park, Durham, NC, United States; ^2^Immune Deficiency Foundation Towson, Towson, MD, United States

**Keywords:** severe combined immunodeficiency, newborn screening, parents and families, information needs, emotional needs, medically underserved, educational materials

## Abstract

**Background:** Now that severe combined immune deficiency (SCID) has been added to newborn screening panels in all 50 states in the U.S., there is a need to develop and disseminate well-designed educational materials to parents who need information to make informed decisions about treatment and care for identified infants. SCID Compass was designed to address this gap. We summarize the results of two needs assessment activities for parents—a journey mapping exercise and online survey—which will inform the development of a website and new resources.

**Methods:** We conducted in-depth interviews with seven parents of children with SCID identified through newborn screening. Participants were asked to complete a journey map to describe key timepoints related to SCID, starting at diagnosis through present day. This qualitative information informed an online survey that was completed by 76 parents who had a child with SCID. All participants were from the United States.

**Results:** Analysis of journey maps revealed five distinct stages that parents experience: (1) Diagnosis, (2) Pre-Treatment, (3) Treatment, (4) Post-Treatment, and (5) The New Normal. At each stage, parents described unique emotions, challenges, contextual factors that can make a difference in their experience, and information and resource needs. Survey results indicated the highest-rated information needs for parents were understanding available treatment options and what to expect across the SCID lifespan. Emotional support needs included dealing with uncertainty about child's future and additional opportunities to connect with other families. Parents preferred receiving new materials from their healthcare provider or other families, and preferred materials in print, from social media, or online. Several differences were found among subgroups of parents, including those whose child had been identified through newborn screening as well as those considered medically underserved.

**Conclusions:** Findings about unmet parent needs and informational preferences will serve as the foundation for creating a suite of resources for those who have a child with SCID. The materials will be tailored to specific stages of the journey. By using a family-centered approach, we will help to ensure that the materials designed and developed as part of SCID Compass will be understandable, comprehensive, and useful.

## Introduction

Severe combined immune deficiency (SCID) is a group of genetic disorders that cause profound defects in cellular and humoral immunity characterized by a deficiency or absence of T-cells (1, 2). Patients with SCID are highly susceptible to severe and recurrent infections, which can result in significant morbidity or mortality in infancy. SCID is considered a rare disease, with prevalence estimates of ~ 1 in 58,000 births (3, 4).

Historically, early clinical diagnosis of SCID was delayed due to a lack of family history or absence of distinguishing symptoms given that infections are common in pediatric populations. However, several studies demonstrated that early identification and provision of immune reconstituting treatment, such as bone marrow transplantation, are highly effective in reducing illness and death in patients with SCID (3, 5–7). Thus, after a robust evidence review by the Advisory Committee on Heritable Disorders in Newborns and Children (8), the United States Secretary of Health and Human Services recommended that all states add SCID and related T-cell lymphocyte deficiencies to their uniform newborn screening panel. As of December 2018, all states have fully implemented newborn screening for SCID. As a result, more children are benefiting from earlier, pre-symptomatic treatment (9).

Despite moving forward with earlier identification, the SCID community continues to face a number of significant challenges, such as a lack of communication between the newborn screening labs, healthcare providers, and families; disparities in knowledge and care for patients with SCID in rural and underserved communities; and generally low awareness and knowledge about SCID and SCID newborn screening for all stakeholders (10). In July 2018, the Health Services and Resources Administration (HRSA) funded a project to directly address the ongoing needs of the SCID community. The goal of the program is to improve the outcomes of infants with SCID detected through newborn screening by increasing awareness and knowledge about SCID; supporting state newborn screening programs; linking families, especially those living in rural and medically underserved areas, to services; and developing long-term follow-up strategies for infants identified through newborn screening. One of the cornerstones of the project, branded SCID Compass, will be a new web site that will house educational materials for parents and all stakeholders.

As part of the family-centered web site development process, the project team wanted to gain a better understanding of unmet educational and support needs and information preferences for parents. However, few studies exist on the information or emotional support needs of parents of children with SCID. A qualitative study of 11 parents of children with SCID reported some common psychosocial stressors and challenges, including the feelings of loss of normalcy and lack of control over multiple aspects of life, the ongoing stress of waiting (both between diagnosis and treatment and between treatment and evidence of treatment efficacy), and the lack of information on how to cope with isolation while in the hospital and after the transition to home (11). Another study suggested that parents of children with SCID who were diagnosed through newborn screening may have higher levels of postpartum depression and posttraumatic stress disorder (12). These findings echo those of a survey of parents who have a child with a rare disease which reported high rates of financial challenges, difficulty in accessing disease-specific support groups, and feelings of isolation, anxiety, and uncertainty (13).

Given the lack of guidance found in the literature, a critical analysis of the challenges faced by parents of a child with SCID was needed. By analyzing the experiences and needs of parents of a child with SCID, we will be able to develop much-needed education materials and ultimately help healthcare providers and the broader newborn screening community to provide more appropriate support and resources. The following questions were addressed as part of the needs assessment:

1. What are the experiences of a parents who has a child with SCID, from diagnosis through post-treatment?
2. What are the informational and emotional support needs of parents of a child with SCID and what are the desired points in time to receive this information and support?
3. Are there any differences in informational and emotional support needs by subgroups of families, specifically those whose child was diagnosed through newborn screening or those who are medically underserved?
4. How do parents rate the available SCID-related materials and what are their preferred sources and formats of materials?

## Materials and Methods

### Design

To answer these questions, we used a two-step, mixed-method approach. First, we conducted a journey mapping activity with a small group of parents who had a child with SCID. A journey map is a diagram or visual representation of a person's experience as they go through a process, often depicted in stages or key points in time (14). As a tool within a user-centered design methodology, journey mapping provides members of the target audience the opportunity to describe their experience from beginning to end, including important milestones, challenges, and successes. Using the qualitative information from the journey mapping activity, we next developed a complementary quantitative survey to expand upon the journey map findings with a broader sample of participants. The goal of both the journey map and the survey was to gain a better understanding of parent's information and emotional support needs and identify opportunities for interventions at key points in time along the journey.

### Participants

Parents were recruited in partnership with the two patient advocacy organizations. The Immune Deficiency Foundation (IDF) and SCID Angels for Life Foundation contacted parents through their Facebook groups, email listservs, and personal calls. Parents who were interested in participating in the journey mapping were referred to study staff to schedule interviews. To be eligible, parents had to be 18 years of age, speak English, and have had a child with SCID identified through newborn screening. A total of seven parents participated in the journey mapping activity.

For the online survey, parents were contacted through similar methods and then directed to the web site to participate. Eligibility criteria for the survey included being at least 18 years of age and having a child with SCID; children did not have to be identified through newborn screening. A total of 76 parents met the eligibility criteria and completed the survey. Table 1 provides an overview of the participants. All participants were from the United States.

**Table 1:** Description of survey participants and their child with SCID.

**Child and family characteristics**	***N***	**%**
Relationship to SCID child		
→ Father	10	13.16%
→ Mother	66	86.84%
Race		
→ White	62	81.58%
→ Black or African American	2	2.63%
→ Asian	1	1.32%
→ Other race	3	3.95%
→ More than one race	6	7.89%
→ Prefer not to say	2	2.63%
Ethnicity		
→ Hispanic or Latino	8	10.53%
→ Not Hispanic or Latino	67	88.16%
→ Prefer not to say	1	1.32%
Live in “Immunology Desert”^a^		
→ No	64	84.21%
→ Yes	12	15.79%
Area description		
→ Urban (large or small city)	34	44.74%
→ Suburban (town outside city limits)	26	34.21%
→ Rural (small community)	16	21.05%
Highest level of education completed		
→ 8^th^ grade or less	7	9.21%
→ Some HS but did not graduate	2	2.63%
→ HS or GED	7	9.21%
→ Some college or 2-year degree	20	26.32%
→ 4-year college graduate	19	25.00%
→ More than 4-year college degree	21	27.63%
Children		
→ Total number of children (Mean, SD)	2.62	1.92
→ Total children with SCID (Mean, SD)	1.28	0.78
→ Children who died from SCID (Mean, SD)	0.20	0.52
→ Age of Child (at time of survey completion)	10.92	10.59
Child's SCID type		
→ Common Gamma Chain of the T-Cell Receptor (XSCID/IL2RG)	32	42.11%
→ Deficiency of the Alpha Chain of the IL-7 Receptor (IL-7Rα)	5	6.58%
→ Deficiency of Janus Kinase 3 (JAK3)	2	2.63%
→ Deficiency of the CD3 Chains	1	1.32%
→ RAG1/RAG2	11	14.47%
→ ADA Deficiency	12	15.79%
→ DCLRE1C (Artemis) Mutation	2	2.63%
→ Other	2	2.63%
→ Unknown	9	11.84%
Child diagnosed with Omenn Syndrome		
→ Yes	2	2.63%
→ No	69	90.79%
→ I don't know	5	6.58%
Diagnosed through newborn screening		
→ Yes	34	44.74%
→ No	42	55.26%

a *Immunology Desert is defined as having a home zip code more than 150 miles from a primary immunodeficiency clinic*.

### Instruments and Procedures

Prior to the journey mapping interview, we emailed participants a worksheet with six empty boxes that fit together to look like a pathway. Using written instructions, we asked participants to think back to the point in time when they first learned their child screened positive for SCID and to select the six points in time when they experienced the greatest successes or challenges related to their child's diagnosis, treatment, and ongoing management of the condition. We also asked about their key information needs and resources they used to meet these needs, and what else would have been helpful. Next, we conducted in-depth interviews using the journey map worksheets to guide the discussion. We asked participants to describe the six time points they selected and share their reasons for including them on their journey map. Approximately 2 weeks after the interviews were completed, we convened all participants in a focus group to review and validate a group-level journey map that we developed based on a synthesis of individual responses. A trained moderator used a semi-structured guide during the interviews and focus groups while a staff member took detailed notes. Participants were offered an incentive to participate in the journey mapping activity.

Approximately 3 months after the journey mapping activities, we launched the online survey. The survey was programmed into Survey Gizmo and was available online in English and Spanish. For those who did not have access to computers or the Internet (e.g., Amish or Mennonite families), a paper version was also available. Printed copies of the survey were mailed to interested participants with a postage paid return envelope. Completed surveys were then entered into Survey Gizmo on behalf of the participant. Data collection was open for about 3 months. The first section of the survey asked questions to gather child and family demographic information. Next, we asked participants to report on their information and emotional support needs. A series of needs were listed; participants were asked whether each was ever a large need, a small need, or not a need. We also asked participants to indicate where they turn to for information about SCID and rate the quality of SCID materials they most recently accessed. Finally, we asked participants about their preferred format and source of materials.

### Data Analysis

The data obtained from the journey mapping interviews were organized using a meta-matrix (15). We then conducted a thematic analysis to identify common stages of a participants' journeys and distill key findings, such as emotional and practical challenges, information needs, and facilitators at each point in time (16). Upon analyzing the seven interviews, we determined that saturation was reached given that participants described similar phases of their journey despite their unique experiences (17). Using findings from the thematic analysis, we created a group-level journey map. We presented the compiled journey map to the interview participants in a virtual group discussion as a validity check.

Survey data were analyzed using SAS Grid Manger, version 7.15 (2017, Cary, NC). Descriptive data include frequencies, means, and standard deviations. In order to calculate mean scores for the informational and emotional support needs items, we scored each need as follows: 0 = Not a need, 1 = A small need, 2 = A large need. For the mean scores that rated the quality of information, we used the following scale: (a) Usefulness: 1 = Not at all useful to 7 = Extremely useful, (b) Ability to understand: 1 = Extremely difficult to 7 = Extremely easy, (c) Ability to find: 1 = Extremely difficult to 7 = Extremely easy, and (d) Trustworthiness: 1 = Very untrustworthy to 7 = Very trustworthy. Parent's rating of their preference for source and format of material was coded as 1 = Not at all interested to 7 = Extremely interested.

In order to examine subgroup group differences, we split parents into dichotomous groups. The first examined differences in needs between those who had a child identified through newborn screening and those whose children were diagnosed clinically. We also wanted to understand any differences in needs between parents who may be medically underserved and those who were not. Thus, we created three different groupings of parents using the race/ethnicity, education level, and geographic location variables as indicators of being medically underserved. Specifically, we defined the groups as (a) non-White or Hispanic parents vs. White, non-Hispanic, (b) those with less than a 4-year college degree vs. those with a bachelors or graduate degree, and (c) those living in a rural area or immunology desert (home zip code more than 150 miles from a primary immunodeficiency clinic) vs. those who were living in an urban or suburban setting and were not in an immunology desert. For each subgroup, parents who met the first criteria (e.g., non-White, Hispanic) were considered medically underserved. *T*-tests were used to detect group differences between mean scores for each of the informational and emotional support needs. Pooled (equal) or Satterthwaite (unequal) test statistics are reported depending on the equality of variances test.

## Results

### Journey Map Findings

**Figure 1:**
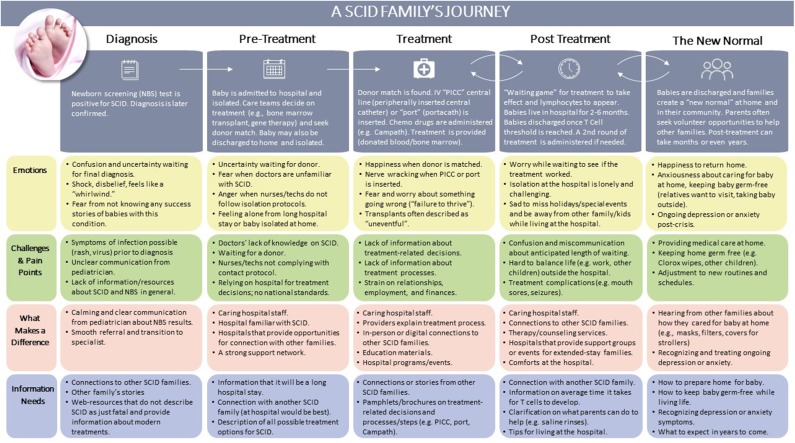
Results of journey map activity.

The goal of the journey mapping activity was to better understand the key experiences of parents of a child with SCID, from initial diagnosis through present day. Analysis revealed five stages that all parents experienced after receiving a positive newborn screen for SCID, as depicted in Figure 1. Each stage is described below, with descriptions of challenges and specific needs at each point in time.

Stage 1: Diagnosis. In this first stage, the infant receives a positive screen and diagnosis is later confirmed. Parents described how their world is turned upside down as they undergo a transition from seeing their newborn as healthy to attempting to understand a surprising diagnosis. The main challenges stem from a lack of readily available, comprehensive resources about SCID and unclear communication from their child's pediatrician or other healthcare providers. However, the provision of clear and useful information from their pediatrician and a smooth referral process to a specialist helped to ameliorate some of these challenges. At this stage, the primary information needs were up-to-date resources about SCID, connections to other families, as well as success stories demonstrating that SCID is treatable.

Stage 2: Pre-treatment. After referral to a specialist, infants are often admitted to the hospital and placed in isolation; they may also be isolated at home. During this time, the healthcare team selects and prepares for a treatment, such as a bone marrow transplant or gene therapy. For bone marrow transplants, typically there is a waiting period while a donor is identified. Parents described challenges around healthcare providers' lack of knowledge of SCID, particularly when nurses or technicians in the hospital do not comply with isolation and contact protocols. Parents also described frustration with the lack of national standards for SCID treatment, noting that treatment decisions are specific to the hospital. Primary information needs in this stage revolved around the benefits and risks of all possible treatments options and getting connected to other families who have gone through the process.

“*Throughout the journey, the bone marrow transplant makes you feel so helpless. If there is no donor what do you do? You just keep waiting. I feel like a lot of education work needs to be done in that area.”*

Stage 3: Treatment. The third stage focuses on the treatment procedure, including conditioning (e.g., chemotherapy). Parents described continuing challenges around the lack of information about treatments for SCID, particularly what to expect during and after treatment. For example, several participants mentioned that there are resources about bone marrow transplants, but almost none of this information is applicable to infants. Available resources often describe transplants for older children or adolescents with leukemia. Parents also described the strain on relationships, employment, and finances as the journey continues several months or more. Parents underscored the need for up-to-date and detailed information about treatment options, processes, and recovery in this stage.

“*His type of SCID is very rare so we didn't know what was going to be the right course of action for him. There was nothing helpful to me. Maybe if parents could be provided brochures with different families' testimonies and stories. I think that would be very helpful for parents going through it now. I didn't know there were so many varieties of SCID and so many treatments for each category. Some can get the newer ones now. I didn't have any of that information.”*

Stage 4: Post Treatment. After their infant received treatment, parents anxiously waited to see if the treatment would be effective. This period can take up to several months, and typically the infant is hospitalized during this process. Parents described the high levels of worry and loneliness during this period, sadness to miss holidays and family events, and frustration at the long duration of the hospital stay. Information needs centered around the anticipated length of time to see if lymphocytes appear, guidance and tips for living in the hospital, and continuing connections with other families who have gone through this process.

“*They were doing bloodwork almost every week. You're waiting for changes but there weren't changes; the levels were still the same. It was hard. It was just a waiting game. Waiting to see when I would be out of the hospital or how long I would be there.”*

Stage 5: The New Normal/Living with SCID. Parents described this stage as the transition back to home and community and living the “new normal.” Parents described a mix of emotions—relief and joy about leaving the hospital and surviving isolation, but anxiety about caring for their child at home and maintaining strict germ-free protocols around their other children, relatives, and during day-to-day life. At this stage, several parents recognized symptoms of depression or anxiety that emerged in this post-crisis period. Others described a need to “give back” in this stage and sought opportunities to volunteer or connect with families beginning their journey. Information needs centered around how to prepare their home for their child after treatment, what to do about ongoing depression and anxiety, and what to expect in years to come.

“*We were officially off isolation and took him to dinner, but I was so nervous to take him out. I wiped the entire table with Lysol and put a mask on him.”*

### Survey Findings

Shortly after the completion of the journey mapping activity, we launched the survey to assess a broad range of informational and emotional support needs. We also asked parents to tell us about their experiences in seeking SCID-related information and their preferred source and format of materials.

Informational support. Table 2 presents means ratings by parents for informational support needs. For all parents, the highest rated informational support needs were: (a) Understanding all available treatment options (1.91), (b) Understanding what to expect across the SCID lifespan (1.89), (c) Knowing what to expect during treatment and hospital stay (1.87), (d) Knowing how to keep child healthy after treatment (1.83), and (e) Knowing where to access specialists (1.79). The lowest-rated information needs were (a) Knowing how to talk to my other children about SCID (0.96), Understanding alternative methods of having another child (1.05), and (c) Understanding the chance of having another child with SCID (1.45).

**Table 2:** Total mean (SD) parent ratings for informational and emotional support needs and by comparison groups.

	**All parents**	**Newborn screening**		**Education level**		**Rural or immunology desert**	
	**Total (*n* = 76)**	**Yes (*n* = 34)**	**No (*n* = 42)**	**Less (*n* = 36)**	**More (*n* = 40)**	**Yes (*n* = 5)**	**No (*n* = 71)**
**Informational Needs**							
Understanding newborn screening results	1.49 (0.81)	1.74 (0.57)	1.29 (0.92)^*^	1.42 (0.84)	1.55 (0.78)	1.60 (0.89)	1.48 (0.81)
Understanding specific type of child's SCID	1.75 (0.59)	1.79 (0.54)	1.71 (0.64)	1.69 (0.67)	1.80 (0.52)	1.80 (0.45)	1.75 (0.60)
Understanding what to expect across SCID lifespan	1.89 (0.39)	1.94 (0.34)	1.86 (0.42)	1.83 (0.51)	1.95 (0.22)	2.00 (0.00)	1.89 (0.40)^*^
Understanding all available treatment options	1.91 (0.37)	1.97 (0.17)	1.86 (0.47)	1.89 (0.40)	1.93 (0.35)	2.00 (0.00)	1.90 (0.38)^*^
Knowing where to access specialists	1.79 (0.50)	1.76 (0.50)	1.81 (0.51)	1.78 (0.59)	1.80 (0.41)	2.00 (0.00)	1.77 (0.51)^***^
Knowing what to expect during treatment and hospital stay	1.87 (0.44)	1.91 (0.29)	1.83 (0.54)	1.92 (0.37)	1.83 (0.50)	2.00 (0.00)	1.86 (0.46)^*^
Understanding managing treatment side-effects	1.72 (0.53)	1.74 (0.51)	1.71 (0.55)	1.81 (0.52)	1.65 (0.53)	2.00 (0.00)	1.70 (0.54)^***^
Knowing how to keep child healthy after treatment	1.83 (0.41)	1.82 (0.39)	1.83 (0.44)	1.81 (0.47)	1.85 (0.36)	1.80 (0.45)	1.83 (0.41)
Knowing where to seek financial assistance	1.58 (0.62)	1.47 (0.61)	1.67 (0.61)	1.69 (0.62)	1.48 (0.60)	1.60 (0.89)	1.58 (0.60)
Knowing how family can adapt	1.64 (0.53)	1.68 (0.47)	1.62 (0.58)	1.61 (0.60)	1.68 (0.47)	1.80 (0.45)	1.63 (0.54)
Understanding the chance of having another child with SCID	1.45 (0.76)	1.56 (0.75)	1.36 (0.76)	1.72 (0.61)	1.20 (0.79)^**^	1.40 (0.89)	1.45 (0.75)
Understanding alternative methods of having another child	1.05 (0.85)	1.26 (0.79)	0.88 (0.86)^*^	1.08 (0.87)	1.03 (0.83)	1.40 (0.89)	1.03 (0.84)
Knowing how to educate family and friends about SCID	1.58 (0.85)	1.53 (0.61)	1.62 (0.62)	1.67 (0.63)	1.50 (0.60)	1.80 (0.45)	1.56 (0.63)
Knowing how to talk to my other children about SCID	0.96 (0.86)	0.97 (0.87)	0.95 (0.85)	1.25 (0.81)	0.70 (0.82)^**^	1.00 (1.00)	0.96 (0.85)
Knowing how to educate hospital staff about SCID	1.49 (0.66)	1.35 (0.65)	1.60 (0.66)	1.47 (0.77)	1.50 (0.55)	1.60 (0.55)	1.48 (0.67)
Knowing how to educate my child's PCP about SCID	1.47 (0.70)	1.38 (0.70)	1.55 (0.71)	1.56 (0.73)	1.40 (0.67)	1.80 (0.45)	1.45 (0.71)
**Emotional support needs**							
Opportunity to talk to other families	1.76 (0.49)	1.79 (0.48)	1.74 (0.50)	1.75 (0.50)	1.78 (0.48)	2.00 (0.00)	1.75 (0.50)^***^
Dealing with uncertainty about child's future	1.79 (0.50)	1.94 (0.24)	1.67 (0.61)^*^	1.69 (0.58)	1.88 (0.40)	2.00 (0.00)	1.77 (0.51)^***^
Managing my emotions as parent/caregiver	1.70 (0.65)	1.85 (0.44)	1.57 (0.77)^*^	1.72 (0.66)	1.68 (0.66)	2.00 (0.00)	1.68 (0.67)^***^
Understanding importance of self-care	1.59 (0.61)	1.68 (0.53)	1.52 (0.67)	1.58 (0.65)	1.60 (0.59)	2.00 (0.00)	1.56 (0.63)^***^
Getting support needed from family or friends	1.57 (0.62)	1.56 (0.56)	1.57 (0.67)	1.69 (0.58)	1.45 (0.64)	1.80 (0.45)	1.55 (0.63)
Managing changes in relationship with partner	1.37 (0.78)	1.44 (0.70)	1.31 (0.84)	1.39 (0.80)	1.35 (0.77)	2.00 (0.00)	1.32 (0.79)^***^
Managing changes in relationship with other children	0.85 (0.91)	0.91 (0.93)	0.80 (0.90)	1.17 (0.92)	0.58 (0.81)^**^	1.20 (1.10)	0.83 (0.90)
Managing changes in relationship with extended family	1.05 (0.79)	1.03 (0.80)	1.07 (0.79)	1.08 (0.84)	1.03 (0.74)	1.60 (0.55)	1.01 (0.79)
Having access to professional counseling	1.32 (0.77)	1.38 (0.70)	1.26 (0.83)	1.22 (0.87)	1.40 (0.67)	1.80 (0.45)	1.28 (0.78)
Having access to professional organizations knowledgeable about SCID	1.74 (0.53)	1.76 (0.50)	1.71 (0.55)	1.61 (0.64)	1.85 (0.36)	2.00 (0.00)	1.72 (0.54)^***^

*0, Not a need; 1, A small need; 2, A large need*.

* *p < 0.05*,

** *p < 0.01*,

*** *p < 0.001*.

When we explored subgroups of parents, we found several noteworthy findings that indicated different informational support needs for those who had a child diagnosed through newborn screening or those who were medically underserved (see Table 2). For parents who had a child diagnosed through newborn screening, there were two statistically significant findings showing higher informational needs than parents whose child was diagnosed clinically: (a) Understanding newborn screening results [t_[69.559]_ = 2.62, *p* = 0.0109] and (b) Understanding alternative methods of having another child (e.g., *in-vitro* fertilization) [t_[74]_ = 2.02, *p* = 0.0488]. When examining differences by education level, parents with less education had more informational needs for (a) Understanding the chance of having another child with SCID [t_[74]_ = 3.19, *p* = 0.00021] and (b) Knowing how to talk to their other children about SCID [t_[74]_ = 2.94, *p* = 0.0044]. Several statistically significant differences were found between those parents who were living in a rural area or immunology desert when compared with those who were not. Specifically, those in a rural area or an immunology desert had more informational support needs for: (a) Understanding what to expect across SCID lifespan [t_[70]_ = −2.38, *p* = 0.0198], (b) Understanding all available treatment options [t_[70]_ = −2.16, *p* = 0.0338], (c) Knowing where to access specialists [t_[70]_ = −3.70, *p* = 0.0004], (d) Knowing what to expect during treatment and hospital stay [t_[70]_ = −2.60, *p* = 0.0114], and (e) Understanding how to manage treatment side-effects [t_[70]_ = −4.57, *p* < 0.0001]. There were no differences in informational support needs when comparing parents who were non-White, Hispanic with those who were not.

Emotional support. As shown in Table 2, the highest rated emotional support needs for all parents were: (1) Dealing with uncertainty about child's future (1.79), (b) Having the opportunity to talk to other families (1.76), (c) Having access to professional organization knowledgeable about SCID (1.74), (d) Managing my emotions as parent/caregiver (1.70), and (e) Understanding the importance of self-care (1.59). The lowest rated items were (a) Managing changes in relationships with other children (0.85), (b) Managing changes in relationship with extended family (1.05), and (c) Managing changes in relationship with partner (1.37).

Emotional support needs also differed by several of the parent subgroups (see Table 2). For those who had a child diagnosed through newborn screening, the following emotional supports needs were rated higher than those who did not have a child diagnosed through newborn screening: (a) Dealing with uncertainty about child's future [t_[55.459]_ = 2.67, *p* < 0.0001], and (b) Managing my emotions as a parent/caregiver [t_[66.869]_ = 2.01, *p* = 0.0489]. Parents who had less education reported that managing changes in relationship with other children was more of a need than those with more education [t_[73]_ = 2.98, *p* = 0.0040]. Several emotional support needs were higher for those who were living in a rural area or an immunology desert, including (a) Having the opportunity to talk to other families [t_[70]_ = −4.28, *p* < 0.0001], (b) Dealing with uncertainty about child's future [t_[70]_ = −3.70, *p* = 0.0004], (c) Managing my emotions as parent/caregiver [t_[70]_ = −4.07, *p* < 0.0001], (d) Understanding the importance of self-care [t_[70]_ = −5.87, *p* < 0.0001], (e) Managing changes in relationship with partner [t_[70]_ = −7.22, *p* < 0.0001], and (f) Having access to professional organizations knowledgeable about SCID [t_[70]_ = −4.40, *p* < 0.0001]. Similar to the informational support needs, there were no group differences in emotional support needs by parents' race or ethnicity.

Information seeking. Parents reported on their most recent experience with seeking information about SCID. Almost a third of participants (30%) reported searching for information within the last 30 days. Another 22% searched for information in the past 1–3 months; 12% in the past 4–6 months, 17% more than 6 months ago, and 18% said they couldn't remember. When asked what topics they were searching for, many parents stated they wanted more information on treatment options, complications related to treatment, or long-term outcomes related to treatments. Others mentioned searching for the latest research while others wanted to connect to other families or were looking for other types of informational or emotional support. Almost half (45%) of parents mentioned that they found the information they were looking for. Over a quarter of parents (28%), though, stated that their search for the specific SCID information they wanted was not successful. The remainder (25%) couldn't remember if they had found the information.

When asked about the quality of the information they had found (*n* = 34), parents rated them as very trustworthy (mean = 6.18, *SD* = 1.24). Slightly lower scores were reported for the usefulness (mean = 5.61, *SD* = 1.25) and ability to understand (mean = 5.55, *SD* = 1.23) the information. The lowest rated dimension was the ability to find the information parents were looking for (mean = 5.15, *SD* = 1.62). White, non-Hispanics were slightly less likely to consider the material that they found as trustworthy than non-White/Hispanics [t_[30.694]_ = 2.37, *p* = 0.0242]. There were no subgroup differences between parents who had a child diagnosed through newborn screening and those who did not, between parents with lower or higher education levels, or between parents who lived in a rural area or immunology dessert and those who did not.

Preferred format and source of materials. Table 3 provides information about parents' preferred formats and sources of materials about SCID. Parents rated receiving information in-person from either healthcare providers or other parents the highest. Reading printed information, interacting on social media, and reading a web site were the next preferred formats. The lowest rated formats were videos and podcasts. Non-whites, Hispanics were more likely to prefer to obtain their information from professionals in person [t_[39.373]_ = 2.18, *p* < 0.0350]. Those with more education had higher preference for podcast [t_[66]_ = −2.25, *p* = 0.0281], social media [t_[56.362]_ = −2.30, *p* = 0.0251], and professional in person [t_[52.593]_ = −2.09, *p* = 0.0416] than parents with less education.

**Table 3:** Total mean (SD) parent ratings for informational and emotional support needs and by comparison groups.

	**All parents**	**White, non-Hispanic**		**Education level**		**Rural or immunology desert**	
	**Total (*n* = 76)**	**Yes (*n* = 58)**	**No (*n* = 18)**	**Less (*n* = 36)**	**More (*n* = 40)**	**Yes (*n* = 5)**	**No (*n* = 71)**
**Preference for material format**							
Professional in person	6.04 (1.25)	5.91 (1.33)^*^	6.50 (0.82)	5.71 (1.51)	6.33 (0.92)^*^	6.09 (1.18)	5.40 (2.07)
Peer to peer	6.03 (1.41)	6.12 (1.33)	5.71 (1.69)	5.86 (1.52)	6.18 (1.32)	6.00 (1.42)	6.40 (1.34)
Print	5.59 (1.53)	5.65 (1.48)	5.38 (1.71)	5.62 (1.72)	5.56 (1.35)	5.57 (1.52)	5.80 (1.79)
Social Media	5.25 (2.09)	5.19 (2.12)	5.50 (1.99)	4.64 (2.37)	5.77 (1.68)^*^	5.16 (2.13)	6.470 (0.89)
Web	5.19 (1.97)	5.26 (1.95)	4.93 (2.09)	4.76 (2.25)	5.55 (1.65)	5.13 (2.00)	6.00 (1.41)
Video	4.77 (1.84)	4.62 (1.82)	5.31 (1.85)	4.51 (2.06)	5.00 (1.61)	4.72 (1.81)	5.40 (2.30)
Podcast	4.40 (1.89)	4.45 (1.89)	4.15 (1.95)	3.83 (2.12)	4.84 (1.59)^*^	4.32 (1.88)	6.00 (1.73)
Other	6.11 (1.05)	6.29 (1.11)	5.50 (0.71)	6.33 (0.82)	5.67 (1.53)	6.00 (1.07)	7.00 (.)

*1 = Not at all to 7 = Extremely*.

* *p < 0.05*.

## Discussion

This paper highlights the journey of parents who have a child diagnosed with SCID through newborn screening and their specific informational and emotional support needs. Our unique design allowed us to gather in-depth qualitative as well as broad quantitative data. It contributes much needed information about the challenges associated with receiving a SCID diagnosis as well as short- and long-term needs. Below, we first note the limitations to our work. Then, we discuss the results of the journey map and survey in context of the current literature. Lastly, we address implications and next steps for SCID Compass in meeting these needs.

### Limitations

There are two main limitations to consider while interpreting these results. First, we used a convenience sample of parents for both the journey map activity and online survey who resided in the United States. By design, the qualitative interviews and focus group used a small, relatively heterogeneous sample of parents. Although the survey had many more participants, it was distributed through the same recruitment channels. Similarly, there were few parents from rural and medically underserved areas. Thus, our findings may not be representative of the entire SCID community, both nationally and internationally. Second, the informational and emotional support needs that parents rated were prespecified based on the data gathered in the journey map. Parents did not have an opportunity to list any additional needs. Therefore, there may be other parental needs that we did not uncover through the survey. Despite these limitations, we believe the are many noteworthy findings.

### Summary of Findings

Parents of children diagnosed with SCID through newborn screening experienced universal elements in their journey, from diagnosis through treatment and finally adjusting to the new normal once they are back home. Key informational and emotional support needs differed throughout the journey. This journey is in stark contrast to the diagnostic odyssey that is typical of clinical diagnosis (18, 19). Others also have noted a distinct journey for those diagnosed though newborn screening, suggesting there are different long-term implications for both parents, their child, as well as different relationships with healthcare providers (20). Our findings also echo the work of Rolland and colleagues that revealed the unique phases of illness as well as parental needs and adaptations that are needed along the course (21).

The highest rated information needs for families were having more information about treatment options, understanding what to expect across the SCID lifespan (i.e., knowing what the journey ahead will look like), knowing what to expect during treatment, and adjusting to life after treatment including knowing where to access to specialists and how to keep their child healthy. Although several studies have examined educational needs of healthcare providers about specific conditions or about newborn screening more broadly (22–25), fewer have focused on the information needs of parents. One study conducted focus groups with parents to better understand what they knew about newborn screening, how they wanted to learn about newborn screening, who should provide the information, and what format would be best (26). An interview study of parent who had received an abnormal metabolic newborn screening result for their child found comparable information needs to those reported in our study, including shock at receiving the test results, frustration in searching for information, and stress related to waiting for confirmatory testing and next steps for treatment (27). Other studies have reported similar information seeking behaviors by parents after a positive newborn screening result (28), such as searching online for information or reaching out to their child's healthcare provider.

Emotional support needs expressed by parents included dealing with uncertainty about their child's future, having access to other families and professional organizations, and understanding the importance of self-care and managing emotions. Earlier work has also reported on parental uncertainty following a positive newborn screen as well as ways to manage and reduce uncertainty (29–31). Common coping strategies include gathering information about the child's condition, assessing the health risk for their child, and seeking support from other. Connecting with other families who have a child with the same condition has been reported by others as well (32, 33). Parent support groups have been shown to provide many benefits, including learning practical skills from other parents; getting relevant, useful information; and obtaining reassurance about the future (34). This can lead to personal growth and empowerment as well as improved emotional well-being.

Our sample of parents who may be medically underserved, which included those who were non-White or Hispanic, those with lower education levels, or those who lived in rural areas or immunology deserts, had even higher informational and emotional support needs across a variety of areas. This included practical needs, such as accessing a specialist, as well as emotional needs, such as navigating relationships. Previous work has highlighted the needs of those who are medically underserved, with particular emphasis on those living in rural areas (35, 36). Other work has shown that education levels and race/ethnicity are social determinants of health (37, 38). The combination of living in a rural area, having lower education, and being a minority can have an additive effect which may result in poorer outcomes for children and their families.

### Implications and Next Steps

Together, these findings suggest high information needs among parents about newborn screening and the implications of having a positive diagnosis for a rare disorder. Not surprisingly, this results in high levels of uncertainty and emotional distress. Thus, parents have an intense need to seek out condition-specific information in order to better understand the impact of SCID on their child and family. Parents also desire to know what the road ahead will look like, including treatment options and how to best care for their child at home. Parents turn to trusted sources for this information, including their healthcare provider as well as other parents who have a child with SCID. Emotional support from other parents can provide hope for the future as well as a way to cope with stress and tend to parent's own mental health needs. For parents living in rural or medically underserved areas, those with lower education levels, or those from racial or ethnic minorities these needs may be exacerbated given the challenges in findings specialists who know their child's condition or other parents who have a child with SCID.

Findings from the parent needs assessment activities will serve as the foundation for creating a suite of resources for those who have a child with SCID. The materials, which will be housed on the SCID Compass web site, will be tailored to specific stages of the journey in order to meet the emotional and informational needs of parents. For example, information about SCID, the different types of SCID, newborn screening, and confirmatory testing will all be directed to parents as they are starting out their SCID journey. Next, during the Pre-treatment, Treatment, and Post-Treatment phases of the journey, parents will be provided information treatment options, the long hospital stay, and how to survive isolation. Information about how to prepare your home and future family planning will be available in the Living with SCID phase of the journey. Emotional support needs, such as where to connect with other parents, how to manage your emotions, and the importance of self-care, are applicable to many stages of the journey and thus will be posted to a dedicated section of the web site. Given that our data show that medically underserved families have increased needs, we will prioritize outreach to them.

By using a family-centered approach to gather data on unmet parental needs, we will ensure that the materials for the SCID Compass web site will be understandable, comprehensive, and useful. Our intent is that the SCID Compass web site will be a vast repository of information for families who have a child with SCID which will meet all their needs and ultimately make their journey with SCID easier.

## Data Availability Statement

The raw data supporting the conclusions of this article will be made available by the authors, without undue reservation, to any qualified researcher.

## Ethics Statement

The studies involving human participants were reviewed and approved by RTI International. The patients/participants provided their written informed consent to participate in this study.

## Author Contributions

MR, LS, HP, and JB designed the overall study. MR, ML, LS, AG, KP, HP, AS, and BF designed the journey map and online survey. ML and AG analyzed the data. MR, ML, and AG drafted the manuscript. All authors critically reviewed the manuscript.

## Conflict of Interest

The authors declare that the research was conducted in the absence of any commercial or financial relationships that could be construed as a potential conflict of interest.
